# Mother-young bond in non-human mammals: Neonatal communication pathways and neurobiological basis

**DOI:** 10.3389/fpsyg.2022.1064444

**Published:** 2022-11-29

**Authors:** Daniel Mota-Rojas, Cécile Bienboire-Frosini, Míriam Marcet-Rius, Adriana Domínguez-Oliva, Patricia Mora-Medina, Karina Lezama-García, Agustín Orihuela

**Affiliations:** ^1^Neurophysiology, Behavior and Animal Welfare Assessment, DPAA, Universidad Autónoma Metropolitana, Xochimilco Campus, Mexico City, Mexico; ^2^Department of Molecular Biology and Chemical Communication, Research Institute in Semiochemistry and Applied Ethology (IRSEA), Apt, France; ^3^Animal Behaviour and Welfare Department, Research Institute in Semiochemistry and Applied Ethology (IRSEA), Apt, France; ^4^Facultad de Estudios Superiores Cuautitlán, Universidad Nacional Autónoma de Mexico (UNAM), Mexico City, Mexico; ^5^Facultad de Ciencias Agropecuarias, Universidad Autónoma del Estado de Morelos, Cuernavaca, Mexico

**Keywords:** bonding, maternal recognition, olfactory, visual, licking, vocalization, sensitive period

## Abstract

Mother-young bonding is a process by which the young establish social preferences for their mother. It fosters reproductive success and the survival of offspring by providing food, heat, and maternal care. This process promotes the establishment of the mother-young bond through the interaction of olfactory, auditory, tactile, visual, and thermal stimuli. The neural integration of multimodal sensory stimuli and attachment is coordinated into motor responses. The sensory and neurobiological mechanisms involved in filial recognition in precocial and altricial mammals are summarized and analyzed in this review.

## Introduction

In mammals, the mother-young bond formation is essential for newborn survival and involves a set of neurological, behavioral, and cognitive mechanisms. Mutual recognition between the mother and the newborn is essential to establish and maintain the bond and guarantee maternal care during the young development (Bolhuis, 2009; Mota-Rojas et al., 2021). The process of mutual recognition between the mother and the newborn involves multimodal sensory signals in the brain of both individuals and occurs during a sensitive period (Broad et al., 2006). This period is important for the mother to recognize and identify their newborn, avoiding misdirected care, reducing energy outlays, and enhancing their reproductive success. Imprinting is a process, mostly studied in birds (Vallortigara and Versace, 2018), by which newborns establish a social preference for their mother, another individual, or even an object (Hess, 1972; Ohki-Hamazaki, 2012). According to Hess (1959), the process of imprinting, observed by Lorenz in European birds, is a phenomenon also reported in other animals such as insects, fish, and some mammals (sheep, buffalo, and deer). However, in the present paper, we will deal with the bonding and interaction process between a young and its mother.

The process of mother-young recognition requires specific sensory stimuli integrated by brain structures (Bolhuis, 1999). It involves structural changes in cortical regions and the release of neurotransmitters that allow newborns to learn to identify their mother—or the individual/object imprinted—through visual, auditory, tactile, and olfactory cues (Sullivan and Wilson, 1994; Insel and Young, 2001; Debiec and Sullivan, 2017). These areas interconnect with other regions to promote the secretion of neurotransmitters associated with the behavioral responses observed during mutual bonding, such as oxytocin, gamma-amino-butyric acid (GABA), glutamate, acetylcholine, and among others (Ohki-Hamazaki, 2012). Neonatal recognition’s set time varies from a few hours to a few days depending on the species. As the mother-young interaction depends on the newborn’s locomotor, sensory, and neuronal level of development, it varies in precocial and altricial species. Offspring from precocial species (e.g., goat kids, lambs, and calves) are born fully developed, have a more developed thermoregulatory system (Muir, 2000; Glatzle et al., 2017), and thus, shortly after birth are capable of following the mother and feed with more independence (Muir, 2000; Glatzle et al., 2017). These species develop discriminative care, allowing exclusive suckling, and a clear chemosensory recognition between the offspring and the dam soon after birth (Horrell and Hodgson, 1992; Booth and Katz, 2000). On the other hand, offspring from altricial species (e.g., canids, rodents, and felines) are not fully developed and have limited sensory and locomotor abilities at birth (Lévy and Keller, 2008), requiring continuous parental protection during the first postnatal weeks, parental grooming, feeding (Scheiber et al., 2017), and in nidicolous species (e.g., rats, mice, rabbits), nesting behavior represents a protective physical barrier, delimiting the space where the mother-young interaction develops (Frohlich, 2020). In many altricial species such as canines, puppies are born with non-functional ears and eyes, and are severely limited in their capacity for mechanical movement (Czerwinski et al., 2016). The cognitive, sensory, and locomotor capacities of both precocial and altricial species are associated with prenatal neurogenesis and degrees of brain maturation (Glatzle et al., 2017).

Complementary, the imprinting process can occur between a young and a human handler, such as observed during artificial rearing in sheep (Markowitz et al., 1998) and sea lions (Lynn et al., 2010). This type of imprinting can be also used as a tool to simplify foal handling (Spier et al., 2004). In these circumstances, the young establish a preferential bond toward the human and activates neuronal pathways related to emotional bond (Coulon et al., 2013). This review aims to summarize and analyze the sensory and neuronal mechanisms of mother-young bonding in non-human mammals.

## Establishment of the maternal-young bond during the sensitive period

The sensitive (or critical) period is a length of time after birth for a mother to become bonded to her young. The formation of this bond depends on maternal responsiveness, social cues from members in the dyad, neuroendocrinological changes, and the display of maternal and neonatal behaviors. The establishment of a mother-young bond implies the social preference of the mother and the offspring for each other. There are several stimuli of distinct origins required for the mother-young bond, that vary according to the development of the offspring’s senses (Bolhuis, 1999; Yamaguchi et al., 2012; Lemche, 2020). At the cerebral level, a key architectural and biochemical configuration gives rise to neuronal circuits, such as the frontal cortex and excitatory neurotransmitters. These circuits participate in creating the neonatal cerebral pathways to establish stable, or preferred, connectivity patterns that act as guidelines for developing a set of behavioral patterns and strategies required for the offspring’s survival (Knudsen, 2004) and even for their adulthood traits, such as sexual behavior (Bateson, 1990). The sensitive period and the structural configuration of brain connectivity depend entirely on the degree of learning achieved. On the other hand, atypical interference can cause irreversible alterations in these brain mechanisms and, therefore, affect the establishment of the bond (Moriceau and Sullivan, 2005). Modifications of neuronal plasticity in the offspring may involve such mechanisms as synaptic consolidation through molecular adhesion cells, abolition of the activation of synapses because of the insertion of stabilizing molecules, and the construction of synapses through the growth of axons or dendrites. In addition, it has been suggested that the development of neuronal networks also requires a certain level of connectivity, or degree of brain maturity. This, however, depends on innate mechanisms, including connections close to the retina or spinal cord. It is important to note that some brain regions involved in the development of the offspring, including the amygdala, cerebral cortex, and hippocampus, maintain a constant level of plasticity throughout life so they can establish long-lasting connection patterns (Knudsen, 2004). In this regard, studies have demonstrated that in numerous mammal species, the primary visual cortex performs a selective elimination of connections in the thalamic axons and that certain actions require the participation of various brain centers (Sullivan and Wilson, 1994; Maekawa et al., 2006;Mora-Medina et al., 2018; Orihuela et al., 2021). Likewise, cascades of molecular elements have been reported to orchestrate the beginning and end of periods of mutual recognition (Sullivan and Wilson, 1994; Insel and Young, 2001; Sullivan, 2003; Moriceau and Sullivan, 2005; Debiec and Sullivan, 2017).

## Sensory channels involved in the mother-young bonding process

This process requires the integration of several sensory inputs that come together from hypothalamic structures, the limbic system, and the cerebral cortex (Mora-Medina et al., 2018; Orihuela et al., 2021). Multimodal signals are transmission pathways that allow learning using sensory stimuli (sight, hearing, smell, taste, and thermal; Glatzle et al., 2017; Mora-Medina et al., 2018; Orihuela et al., 2021). The main involved pathways are conditioned by the degree of sensory functionality characteristic of the offspring at birth (Mora-Medina et al., 2018). Dams can recognize auditory, visual, and odor signals, but newborns are conditioned by the degree of their sensory development, so they are incapable of capturing certain features of their physical and social environment until their senses and communication channels reach optimal levels of development (Muir, 2000; Werneburg et al., 2016). In altricial species, olfactory cues are essential for the onset of young-mother interaction after parturition (Poindron et al., 1988; Lévy and Nowak, 2017), and communication is practically unidirectional (Muir, 2000). For instance, rat pup-dam licking behavior is regulated by the pup’s preputial gland secretion (Brouette-lahlou et al., 1991b). However, some altricial neonates, including those of feline species, can detect odor stimuli that help them locate the mother’s mammary gland (Vitale, 2018). In mice, Al Aïn et al. (2013) elegantly demonstrated that mouse pups imprint on their own mother’s odor *in utero*, and these imprinted odors from the amniotic fluid improve nipple attachment of the pups by inducing nipple grasping (Workman et al., 2013). The development of neural structures in the offspring is probably related to acquiring a more functional sensitive sensibility (Workman et al., 2013; Glatzle et al., 2017).

On the other hand, the offspring of precocial species can establish communication channels only a few minutes after birth, as they can perceive sensory stimuli and perhaps even follow the mother due to their fully-developed motor neurons (Workman et al., 2013). This occurs, for example, in water buffaloes, bovine cattle, goats, and sheep. These four precocial species are classified as prey animals that are in constant movement; therefore, the young and the mother must have the ability to identify each other immediately after birth, especially in the case of large social groups (Glatzle et al., 2017; Mora-Medina et al., 2018; Orihuela et al., 2021). Overall, the communication pathways depend on the degree of neurodevelopment of the neonate (Mora-Medina et al., 2018; Orihuela et al., 2021).

### Tactile and thermal stimuli

Tactile stimuli serve to form the mother-young bonding and contribute to the offspring standing, finding the teat/udder to feed, and suckling (Vince, 1987; Vázquez et al., 2015).

Numerous studies have highlighted the importance of the licking on the anogenital region and other areas of the newborn’s body because it can influence emotional responses from the mother, as observed in dogs (Lezama-García et al., 2019). In lambs, a precocial species, tactile stimulation is the primary stimulus that the offspring receives (McGlone and Stobart, 1986). It also reduces stress and promotes the development of social skills in neonates, as has been demonstrated in rats (Caldji et al., 1998; Champagne et al., 2003; Starr-Phillips and Beery, 2014). Licking the face encourages the newborn to stand and start mouth and munching movements resembling suckling, particularly when the head of the newborn touches warm and hairless areas such as the udder (Mottershead et al., 1985).

In this aspect, the differences in the skin temperature of the dam play a key role in teat-seeking and the start of suckling (Vince, 1993). Authors such as Vince (1984) found that lambs prefer warmer naked surfaces between 32 and 39°C such as the inguinal area and the udder (around 35°C–37°C). The average value of 36°C influenced the suckling time (55.6 ± 6.62 s) and the maintenance of the contact between the nose and lips. Likewise, neonatal pigs use thermal and tactile cues as motivation for teat-seeking (Hrupka et al., 2000). Nonetheless, this reaction does not only depend on thermal influence since other cues are necessary (Vince, 1984).

The endocrine influence of estradiol and progesterone has been tested by Le Neindre et al. (1979) and injection of ovarian steroids influences licking behavior and selective recognition in the mother. All this has beneficial responses in the young, including diminished anxiety (Meaney, 2001), but also has long-term consequences, as the presence/absence and frequency of licking modify the sensitivity of the newborn brain to different hormones in adulthood (Meaney, 2001). However, whether there are differences in these responses among altricial or precocial species remains to be studied.

### Visual stimuli

The visual communication pathway participates in the mutual recognition and bonding of the offspring to their mother. In altricial neonates, however, the uptake of visual stimuli is delayed because the development of retinogenesis and corticogenesis occurs gradually after birth (Workman et al., 2013; Glatzle et al., 2017).

The ability of a newborn to detect visual stimuli depends on the species and different levels of functional maturation. Moye and Rudy (1985) determined that 15-days-old rat pups are able to detect visual and auditory stimuli; however, they cannot associate visual cues until day 17th, due to the morphological differentiation of the visual cortex neurons happening around postnatal day 21 (Miller, 1986). Additionally, although eye opening is a key process for any animal, in rats, before pups open their eyes, head direction cells of the hippocampal formation and anterodorsal thalamic nucleus integrate sensory information and appear 3 days before eye opening, going through a rapid maturation after this stage (Tan et al., 2015). Cancedda et al. (2004) demonstrated that maturation and ability to process visual stimuli can be affected by the sensory-motor environment in mice, where newborn pups reared in enriched environments (wire mesh lid, running wheel, tunnels, shelters, stairs, among others) caused a precocious eye opening (71% at day 12 in enriched animals vs. 6% in the control group), a faster visual acuity development by 6 days compared to control animals, and this also had an influence in maternal care, since high concentration of brain-derived neurotrophic factor (BDNF; a neurotransmitter found in enriched animals) are associated to higher levels of licking.

### Olfactory stimuli

Olfaction has an essential role in the modulation and establishment of the dam-offspring bond in several mammal species (Lévy et al., 2004). For example, the olfactory cues derived from the preputial gland of rodent pups (dodecyl propionate), are associated with the beginning of licking in this area (Brouette-lahlou et al., 1991a). Olfactory signals are detected by the main olfactory system, composed of the main olfactory epithelium (MOE), of which olfactory neurons project to the olfactory bulb (OB). However, the requirement of full functionality of the OB in some species is not necessarily associated with behavioral responses of the newborns, as nipple attachment or suckling are not affected in seven-day-old rat pups with partial bulbectomy (Risser and Slotnick, 1987). Similarly, newborn rabbits with a medial or lateral removal of 80% of the OB still responded to pheromones and odor signals without altering their suckling behavior (Hudson and Distel, 1987), reinforcing the concept that partial functionality might be enough at this stage of development.

Indeed, some “olfactory” cues such as the pup’s preputial gland pheromone are detected by the vomeronasal organ (VNO), forming with the Accessory Olfactory Bulb (AOB) the Vomeronasal System (VNS) or Accessory Olfactory Sytem (AOS; Brouette-Lahlou et al., 1999). In addition, some authors demonstrated that the maternal VNS is essential for the early mother-infant bond in the rat (Del and Cerro, 1998) or for the neonatal offspring recognition in sheep (Booth and Katz, 2000). On another hand, the mammary pheromone can enhance olfactory learning in rabbits pups by functioning as a “cognitive organizer” that promotes early learning of environmental cues (Coureaud et al., 2006). Thus, this could help the rapid development of mother-young recognition and bonding.

### Auditory stimuli

The role of auditory perception of the offspring has been still little studied in mammals. The hearing ability observed in animals at birth mainly depends on whether it is an altricial or precocial species. For example, guinea pig pups (a precocial species) can hear and respond to auditory cues at birth (Ehret, 1980). Similarly, newborn lambs between the second and 35th postnatal days do not have a significant difference in latency or amplitude of brainstem auditory evoked responses, due to the prenatal maturation of the brain in precocial species (Ashwal et al., 1984).

Contrarily, rat pups are deaf at birth and their auditory cortex evokes responses only after the first 2 weeks post-partum (Makarov et al., 2021). In neonatal rabbits, thalamocortical axons are the main sensory afferent before hearing onset and can be found as early as postnatal day one (de Venecia and McMullen, 1994); however, rabbit pups cannot hear until day 7, approximately (Kral and Pallas, 2011). Additionally, all newborns from any species can produce the “isolation call,” a vocalization present when exposed to distressful environments (Ehret, 1980). When rat pups are isolated from their mothers, ultrasonic vocalizations occur mainly during the first 6 to 7 days postpartum and elicit searching or retrieving of the pups (Nagasawa et al., 2012).

Another example is the female Australian sea lion, which develops a rapid bonding with its pup within a few days after parturition since it has to return to foraging sea very shortly after. This bonding is primarily mediated *via* vocal recognition as both mothers and pups produce individually stereotyped vocalizations, named attraction calls (Charrier and Harcourt, 2006; Charrier et al., 2022). Identity is encoded in amplitude modulations, frequency modulations, and frequency of call (Pitcher et al., 2012). Then, mother recognition of pup calls is established within 48 h of birth, before the female leaves to forage (Pitcher et al., 2010), but pups cannot identify their mother’s voice before the end of the perinatal period, during their first separation from their mothers (Pitcher et al., 2009). Pups only play a more active role at a more advanced period when they have acquired their vocal discrimination abilities.

## Neural circuits involved in the mother-young mutual recognition process

### Neural changes during tactile recognition

**Figure 1:**
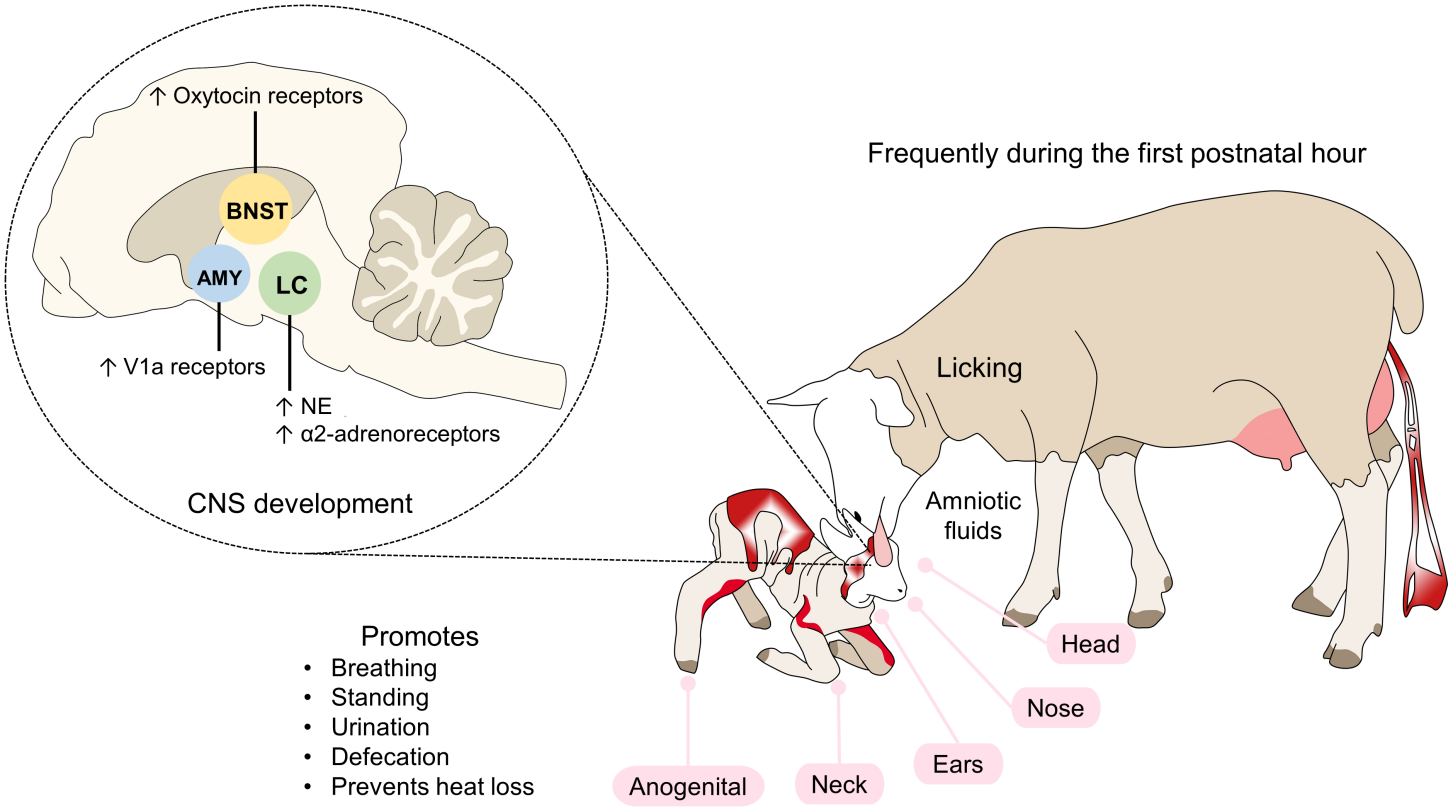
Tactile recognition in sheep. Tactile cues include licking the newborn, particularly during the first hour of life. By starting at the head, nose, ears, and the anogenital region, the mother encourages the offspring to stand up and consume colostrum. The endocrine response in lambs activates zones in the LC, AMY, and BNST, where an increase in noradrenergic neurons, adrenergic, oxytocin or V1a receptors occurs. AMY, amygdala; LC, locus coeruleus; NE, norepinephrine; V1a, vasopressin 1a.

All tactile stimuli activate the locus coeruleus (LC), which has the largest reserve of norepinephrine (NE) and is the brain region that provides this neurotransmitter to the olfactory bulb (Debiec and Sullivan, 2017). Licking activities depend on the action of the hippocampus and limbic system with participation by the amygdala and nucleus accumbens (Figure 1; Francis et al., 2002; Lévy and Nowak, 2017; Orihuela et al., 2021).

In rodents, licking newborns affect the expression of glucocorticoids receptors in the hippocampus (Liu et al., 1997), oxytocin and vasopressin receptors in the central nucleus of the amygdala, bed nucleus of the stria terminalis, and lateral septum (Francis et al., 2002; Curley et al., 2012), and estrogen receptors in the medial preoptic area (Champagne et al., 2003). Maternal contact also increases the cerebral oxytocin concentration of the offspring (Kojima et al., 2012).

In addition, in a dopamine genotype-dependent manner, female rat offspring that received higher early-life licking displayed higher dopamine levels in the nucleus accumbens and provided higher late-life licking to their pups (Lauby et al., 2021). Likewise, differences in estrogen receptors expression in the medial preoptic area (MPOA) are transmitted from the mother to her female offspring and influence their licking/grooming behavior of pups at the adult age (Champagne et al., 2003).

### Neural changes during visual recognition

The process of visual imprinting has been studied in depth in birds (Beecher et al., 1981; Bolhuis, 1991; Maekawa et al., 2006; Nakamori et al., 2013), although there are few studies in mammals. Previously, it was stated that the level of neurodevelopment of mammals at birth influences their ability to recognize the mother and the environment through their eyes. In general, in young mammals, the visual system is the last to develop, just after auditory, proprioceptive, vestibular, and nociceptive systems (Mellor, 2019). For example, in lambs, the visual cortex at birth has a similar physiology of adults (Clarke et al., 1979), which helps in recognizing the mother in the first minutes post-partum (Mellor and Diesch, 2007). Visual stimuli travel through the optic nerve and link to the occipital lobe and lateral geniculate nucleus. Signals are projected to the visual cortex, which senses and then establishes visual configurations that allow mutual identification between the dam and the offspring at a distance (Mora-Medina et al., 2018; Orihuela et al., 2021).

In birds, the region analogous to the mammalian visual cortex is called the visual Wulst. Studies of this region using optical imaging techniques show that imprinting on a specific object increases the activity of the neurons of this area, as well as their synapses (Maekawa et al., 2006). Upon receiving signals from the retina, the visual Wulst transmits the information to the posterior region of the telencephalon, the nucleus rotundus in the thalamus and entopallium, the core and periventricular regions of the hyperpallium densocellulare, and the intermediate medial mesopallium (IMM; Ohki-Hamazaki, 2012). The IMM is a zone where early learning derived from visual stimuli is consolidated (Insel and Young, 2001). In newly hatched chicks, the IMM also receives afferents from the optic tectum, hippocampus, amygdala, and regions of the nidopallium (Nakamori et al., 2013). The information processed there is transmitted to the amygdala and, subsequently, the medial striatum (MSt) and its dopaminergic nuclei (Nakamori et al., 2013).

**Figure 2:**
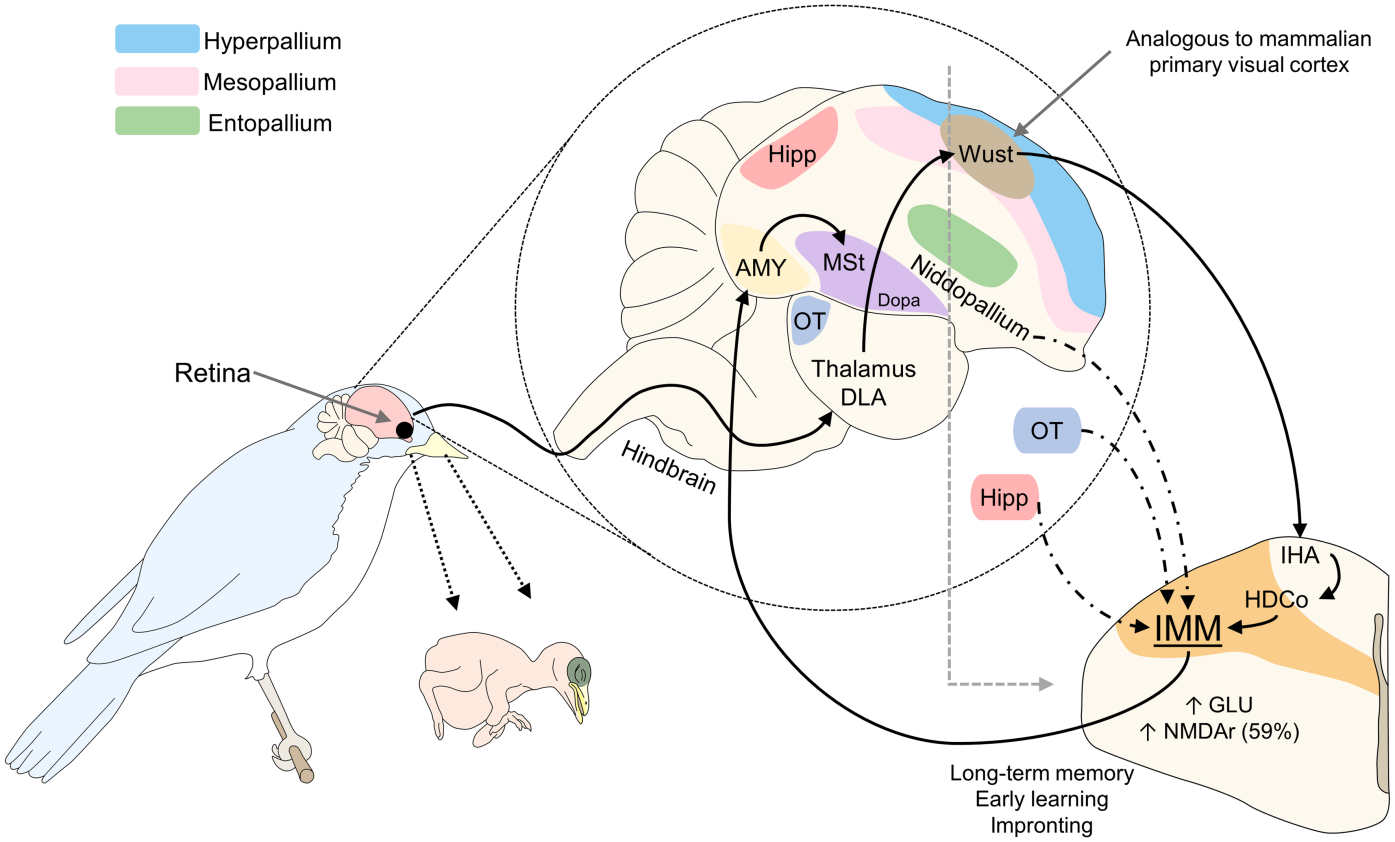
Visual imprinting in birds. The stimulus captured by the retina is projected to the thalamus DLA and Wust, a region of the hyperpallium analogous to the mammalian primary visual cortex. Through connections to the IHA and HDCo, visual signaling reaches the IMM, the main structure involved in long-term memory, early learning, and imprinting. During the sensitive period, amounts of glutaminergic neurons and NMDAr in this zone increase to promote imprinting. AMY, amygdala; DLA, dorsolateralis anterior thalami; Dopa, dopamine nuclei; GLU, glutamate; HDCo, hyperpallium densocellulare; Hipp, hippocampus; IHA, hyperpallium apical; IMM, intermediate medial mesopallium; MSt, medial striatum; NMDAr, N-methyl-D-aspartate receptors; OT, optic tectum.

After hatching, birds show an increase in excitatory glutamatergic transmission in the IMM that remains high for at least 24 h (Meredith et al., 2004) and an increase in spinal excitatory synapses of the IMM (McCabe, 2019). Key neurotransmitters involved in this process are NE, glutamate, acetylcholine, GABA, and taurine. Also, the density of N-methyl-D-aspartate receptors (NMDA) shows high activity and plasticity in the IMM (Nakamori et al., 2013). It has been reported that during imprinting the density of NMDA receptors increases by more than 59% on the left side of the IMM, an area associated with long-term memory (Figure 2; Bateson and Horn, 1994; Bolhuis, 1999; McCabe, 2013). Nakamori et al. (2015) found that NMDA receptors containing a specific subunit (NR2B/NR1) are expressed at the beginning of the imprinting process and the knockdown of these subunits impairs imprinting. Likewise, McCabe and Horn (1994) determined that the expression of the early gene Fos in the IMM of chicks participates in the learning and memory process of imprinting.

An interruption in the functioning of these receptors has been associated with failed imprinting in precocial birds such as ducks, chickens, and geese (Ohki-Hamazaki, 2012). The lack of stimulation during the sensitive period generates structural alterations in the brain that result in a deprived visual cortex (Hubel et al., 1977), alterations of the auditory cortex, and structural changes in the olfactory bulb (Wong-Riley, 2021). In the case of mammals, blindfolded lambs did not stand in the first hours of life and did not approach the dam, showing that visual stimulation is important to establish the maternal bond (Vince et al., 1987).

During visual imprinting, the plasticity of the visual cortex depends on this sensitive period and is not altered by external effects (Bischof, 1983). The effects of cortical plasticity are seen during the first few weeks of life in the offspring. For example, cats’ dendritic spines increase during the first 8 weeks of life, though their number decreases at the end of the sensitization period (Bischof, 1983).

### Neural changes during olfactory recognition

**Figure 3:**
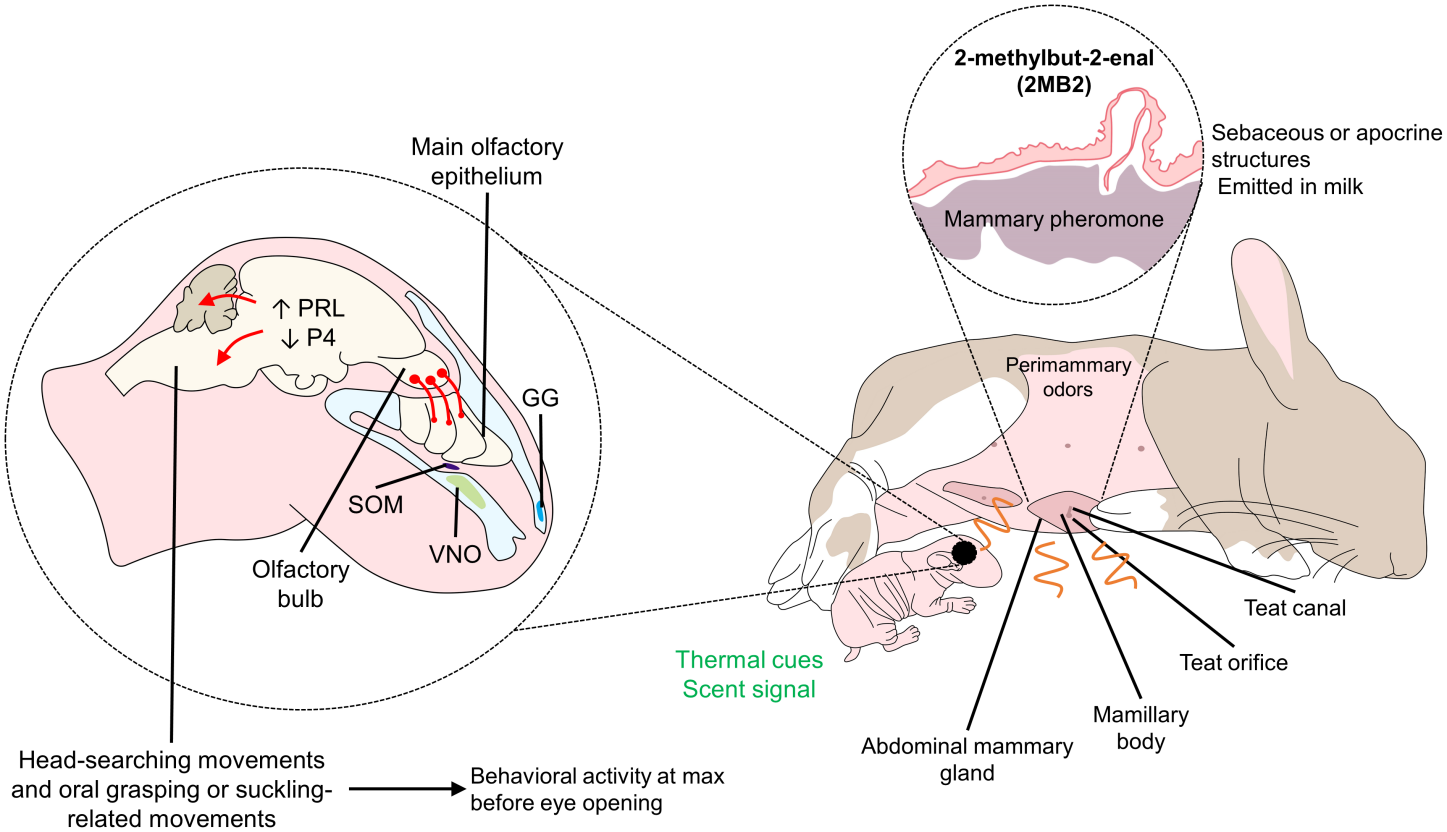
Olfactory signaling and processing in rabbit pups. Although pups cannot see at birth, perimammary odors are responsible for olfactory signaling in newborns. The mammary pheromone (2 MB2), a pheromone presents in sebaceous structures and released in the milk, is known to incite the head-searching and oral grasping movements of the newborn. Rabbit pups can detect the pheromone through peripheral (VNO) and central (olfactory bulb) structures of the auditory system, promoting an endocrine response that contributes to behavioral changes. GG, Grueneberg ganglion; P4, progesterone; PRL, prolactin; SOM, septal organ of Masera.

Olfactory recognition of the mother and odor stimuli are essential for neonate animals whose visual systems are not completely functional at birth, as occurs in altricial species (Leon, 1992), in whom tactile input has a stronger association with attachment (Nowak, 2006). Indeed, early olfactory associative learning (wherein an odorant conditioned stimulus is temporally paired with another unconditioned stimulus to produce a conditioned behavioral response to the odorant) must occur for the pup to survive weaning (for instance, in the nest, for nipple attachment and closeness to the mother). Consequently, conserved neural mechanisms under strong evolutionary control are involved (Sullivan and Wilson, 2003). For example, in lambs, neurons at the olfactory bulb (OB) are continuously generated and in the first developmental stages, these neural cells rapidly divide into neuroblasts to migrate and form interneurons, involved in the recognition of environmental odor cues (Corona et al., 2018). The synapsis of OB interneurons in the embryonic brain is mediated by the inhibitory transmitter GABA that contrarily to its main activity, has an excitatory nature in neonates, as Carleton et al. (2003) reported in mice. In addition, this process was shown to rely on relatively reduced neural circuitry. In rats, areas like the amygdala, hippocampus, and frontal cortex are not fully developed at birth, so those newborns require other pathways for learning and bonding. This process depends on the activation of nerve terminals in the OB, LC, and olfactory cortex, involving neurotransmitters like NE and serotonin (Yuan et al., 2003; Debiec and Sullivan, 2017), which has an essential role during the sensitive period (Figure 3; Hussain, 2011; Johnson-Delaney and Orosz, 2011; Mucignat-Caretta et al., 2012). In particular, the OB and accessory OB are the principal structures involved in olfactory mother-young recognition in mice and rats (Hudson, 1993), also with a central role of noradrenergic afferences to these areas. In particular, the blockage of beta-noradrenergic receptors inhibits the olfactory preference process (Shakhawat et al., 2012). The NE reduces the inhibitory action of GABA, thus strengthening their synapses, and acts on the β1-adrenoreceptors of mitral cells in the olfactory bulb, participating in the learning of the maternal odor, thus fostering neural plasticity (Yuan et al., 2003; Debiec and Sullivan, 2017). In this species, the increased function of noradrenergic neurons in the LC and the hypofunction of the amygdala facilitate preference learning while blocking odor aversion (Sullivan, 2006). The continuous activation of mitral cells in the OB causes metabolic changes and promotes learning odors (Sullivan, 2006). Similarly, the duration of the sensitive period (7–10 days) coincides with the presence of noradrenergic locus coeruleus neurons in the OB, which acquire adult characteristics in the same period (Debiec and Sullivan, 2017). Odor and tactile stimulation also cause an increase of 400% in extracellular dopamine concentrations (Leon, 1992).

**Figure 4:**
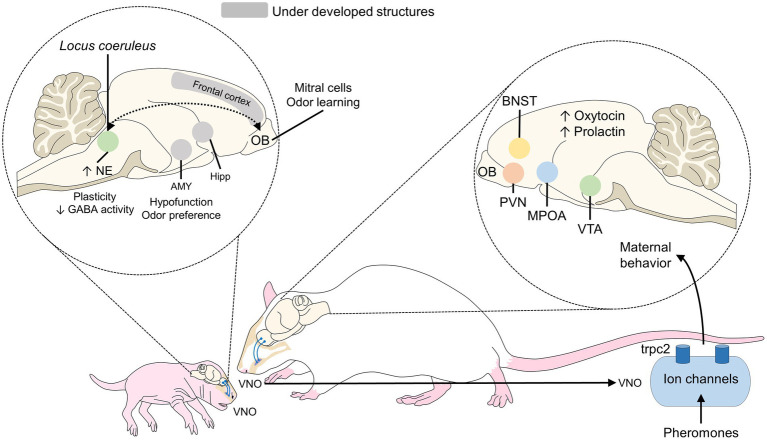
Olfactory mother-young recognition in rodents. During this process in neonate rats, the hypofunction of the amygdala (to block odor preference) and the greater number of noradrenergic neurons in the locus coeruleus are the main features that promote the interaction. In mothers, oxytocin secretion and its action on the MPOA, VTA, PVN, and BNST are associated with the presentation of maternal behaviors. AMY, amygdala; BNST, bed nucleus of the stria terminalis; Hipp, hippocampus; GABA, gamma-aminobutyric acid; MPOA, medial preoptic area; NE, norepinephrine; OB, olfactory bulb; PVN, paraventricular nucleus; VTA, ventral tegmental area.

Hippocampal activity has been studied during mother-young bonding in sheep. Findings show that the number of maternal cells that respond to odor stimuli from lambs increases by approximately 60% in the first weeks postpartum; that is, during the sensitive period so the olfactory recognition is facilitated. Around half of those cells respond only to filial offspring. Likewise, increased GABA and glutamate concentrations are observed in the OB. In the case of neonates, as shown in Figure 4, the reduced plasticity to GABA, hypofunction of the amygdala, and other underdeveloped structures such as the frontal cortex, hippocampus, and amygdala, participate, together with the OB, in odor learning (Insel and Young, 2001).

### Neural changes during auditory recognition

There is scarce information on the neural changes related to auditory cues during mother-young interaction, although some authors refer to this type of recognition as more relevant than visual (Bolhuis and Van Kampen, 1992). The main structures involved in auditory stimulation are the auditory cortex and forebrain, but activity in the dorsal region of the cerebral hemispheres and the roof of the forebrain predominates (Orihuela et al., 2021). In chicks, the hyperstriatum accessorium, lateral neostriatum, and medial neostriatum/hyperstriatum ventrale respond to 1.8-kHz sounds (Leon, 1992). Specifically, the mediorostral neostriatum responds to auditory stimuli (Insel and Young, 2001).

Wolfson et al. (1990) found that in premature lambs, brain-stem auditory evoked potentials are present at 106 days of gestation, and fully mature at within the first 122 days of gestation, making this a sensitive developmental stage. In contrast, in neonatal rats, the auditory function begins on post-natal day 12–14 and until day 22, the hearing function is similar to an adult (Geal-Dor et al., 1993). In another rodent, Octodon degus, “mothering calls” incite a metabolic activity in the somatosensory frontoparietal and frontal cortex of two-week-old pups. In these animals, Braun and Scheich (1997) reported that the pups can create an association between the vocalizations and the proximity of the mother, contributing to the process.

## Importance of the role of neuromodulators

During mother young interaction, communication networks induced by essential neurotransmitters develop. These networks favor the learning process of the newborn (Castro-Sierra et al., 2007; Orihuela et al., 2021). The neuropeptides involved show activation of the hypothalamus, which secretes hormones and neurotransmitters (Insel, 2010). Several neuromodulators are involved in the mutual recognition, including oxytocin, GABA, glutamic acid (GLU), monoamines such as dopamine (DA) and serotonin (5-HT), as well as N-methyl-D-aspartate (NMDA), prolactin (PRL), and BDNF (Castro-Sierra et al., 2007). For example, newborn rats should learn their mother’s odor, orient to her, select a nipple and huddle to favor its’ development (Teicher and Blass, 1977). Nevertheless, this system improves both learning and attachment while pups are confined in the nest (Sullivan, 2003; Moriceau and Sullivan, 2005). In this attachment process, there are three important structures involved: the olfactory bulb, the noradrenergic locus coeruleus, and the amygdala (Sullivan, 2003).

Oxytocin is a hormone primarily synthesized in magnocellular neurons of the PVN and supraoptic nuclei (SON) of the hypothalamus. Its connection to the posterior pituitary, where it is stored as secretory vesicles, causes its release into the bloodstream. In addition, the dendritic release of oxytocin into the extracellular space through the brain has local effects on specific tissues and biological functions. Furthermore, smaller parvocellular neurons in the PVN also produce oxytocin and have connections to other brain structures including the limbic system (notably the amygdala and hippocampus) and the nucleus accumbens. Hence, oxytocin can also act as a neurotransmitter/neuromodulator. There is also a positive autoregulation action of oxytocin itself (Meyer-Lindenberg et al., 2011). Besides, it is influenced by gonadal steroids that foster its synthesis and modulation (Sharpey-Schafer, 1933; Orsucci et al., 2013). Oxytocin binds to the oxytocin receptor present in several cerebral regions, suggesting that the oxytocin receptor in the central nervous system has a wide variety of effects (Jurek and Neumann, 2018).

This neurotransmitter is involved in the mother-young bonding notably *via* the mediation of mother preference as shown in infant lambs treated with synthetic antagonist OT receptors which led to a decrease of the exploration of the mother’s body and impaired the expression of the mother preference (Nowak et al., 2021). Furthermore, these authors also showed that close social contact of the young with its mother during suckling periods (probably through somatosensory stimulations from the orogastric sphere) triggered the release of OT in the lamb’s plasma and cerebrospinal fluid, as also previously described in calves’ plasma (Lupoli et al., 2001).

PRL is a polypeptide neurohormone that plays multiple homeostatic roles in the organism in conjunction with the dopaminergic and oxytocinergic system, since PRL secretion is controlled by a complex network of positive (oxytocin) and negative (dopamine) feedback loops (Freeman et al., 2000; Kennett and McKee, 2012). PRL receptors on the MPOA participate in the initiation of maternal recognition (Salais-López et al., 2021), the development of neural systems that underline the control of maternal behavior, as well as immune and reproductive development of the offspring, as studied in rat pups deprived from maternally-derived PRL intake through the milk (Melo et al., 2009). This hormone and its role on the fetal brain and activation of neural circuits that trigger maternal behaviors when adults has been studied in mice pups, where the lack of receptors to produce PRL results in normal pups grow but nursing deficiencies when reaching adulthood (Sairenji et al., 2017). In addition, the expression of PRL receptors at all levels of the olfactory system of rat’s neonates indicates that PRL participates in the differentiation and development of the olfactory system. Thus, in the neonatal period, PRL may modulate olfactory function that plays a key role in the interactions between the newborn pup and its mother (Freemark et al., 1996).

Another example is BDNF, a transmitter that modulates the plasticity of the visual cortex in rodents during early postnatal development, together with GABAergic neurons. This is relevant because studies have shown that a higher concentration of these markers promotes an active interaction with their environment, including the mother, enhancing the level of licking in these pups and, consequently, a precocious maturity of the visual system (Cancedda et al., 2004).

## Future directions

In intensive production systems, neonates and dams are frequently separated shortly after birth, truncating the mother-young bonding process, and its associated neurobiological developments that can reduce the welfare of both mother and newborn, including until later at the adult age. Hence, an especially important field for the future study includes the development of new procedures that do not impact the performance of production units but benefit animal welfare. In that sense, it is important to understand the processes in different species to propose new production models that enhance the current practices. New research perspectives are opening, focusing on analyzing the neurobiological processes inherent to the mother-young recognition, where pheromonal communication also plays an important role. However, although this kind of mediation has been studied in female rodents (Larsen et al., 2008), the role of maternal pheromones on the neonatal brain is still a research field that could be investigated thoroughly.

The implication of mother-young mutual recognition and parenting behavior on the maternal behavior of the newborn in their adulthood is also of interest, where studies in rodents have shown that oxytocin release and an adequate mother-young dynamic promotes enhanced maternal care for future generations (Nagasawa et al., 2012). Likewise, social behavior is an important aspect in breeding of farm animals, notably the agonistic interactions. In that sense, Toinon et al. (2022) showed that lambs reared with or separated from their mother displayed different social behaviors after weaning and mixing, with dam-reared kids initiating more but receiving less agonistic interactions the dam-separated kids.

Future research could also aim to qualify the particular effect of mother-young attachment on the young’s brain and whether the term imprinting could or could not apply to mammals as a notion of “brain imprinting” or “neurobiological imprinting” to describe the impact of the maternal bond on brain circuits and functioning on the later offspring phenotype, as it is already done in genetics with the term “genomic imprinting” found in mammals to describe the influence of a particular parental allele on offspring’s gene expression and phenotype (Tucci et al., 2019).

Imprinting is a process that is not exclusively created between the mother and the neonate but can be observed in newborns and humans. For example, imprint-trained foals to a human tactile stimulus (e.g., gentle rubbing) at birth decreased the defensive behaviors of the same animals at 3 months of age, facilitating their handling (Spier et al., 2004). A similar case was observed in lambs of 1- to 9-days old who were fed and handled by humans reduced avoidance behavior (Markowitz et al., 1998). However, some other reports on foals mention that early exposure to humans does not have significant differences in foal behavior at haltering and handling after 6 months (Williams et al., 2003), and even the cases where human aid on the first suckling causes an evasion response at 1 month (Henry et al., 2006).

In contrast to the importance of imprinting in domestic animals, in the area of wildlife rehabilitation, a fundamental requirement for the successful release of animals into their natural habitat consists, precisely, of preventing imprinting (Perry and Averka, 2020). Lynn et al. (2010) reported in a hand-reared sea lion that filial imprinting can cause disrupted social behaviors, where the sea lion needed to be returned to captivity since the animal responded to human voice and seek human presence following release. To prevent this, wildlife rehabilitation and reintroduction programs use techniques that prevent contact between animals and humans to impede the development of attachment (Royal Society for the Prevention of Cruelty to Animals, 2010). For example, “costume-rearing” is used with young animals in whooping cranes in reintroduction programs (Olive and Jansen, 2017). Imprinting processes and the responses they generate depend not only on species but also on the purpose for which animals are raised.

## Conclusion

Mother-young bonding is an essential process for establishing the relationship between offspring and the dam. Auditory, olfactory, tactile, and visual stimuli participate in the onset of bonds through cerebral structures like the LC, with a key role of NE concentrations. During the sensitive (critical) period, the newborns’ brain is highly receptive to their mothers’ stimuli, a feature that is an essential component for affective bonding and the learning of information necessary for survival. To trigger this mechanism, structures such as the olfactory bulb, auditory cortex, visual cortex, locus coeruleus, and some areas of the limbic system are activated. They communicate through neurotransmitters that promote early learning in the newborn and maternal behaviors in the dam. Neonatal recognition of the mother thus depends on a whole series of neurobiological processes that begin with the uptake of multimodal sensory stimuli, which promote the formation and development of communication channels initiated by neurotransmitters. Oxytocin has been described as the principal neurochemical substance associated with affiliative and learning behaviors through conditioned affective associations that become integrated into motor responses.

Finally, it is important to emphasize that although the bonding mechanisms promoted by the aforementioned stimuli are typical of both precocial and altricial animals, differences among them may make one mechanism more important than others during the first days of life; for example, olfactory interaction in rodents. Learning more about these characteristics will help ensure that initial contact and response of non-human animals contribute to neonatal survival by promoting or preventing bonding, depending on the species.

## Author contributions

DM-R, CB-F, MM-R, AD-O, PM-M, KL-G, and AO contributed to the conceptualization and writing and reading of the manuscript. All authors contributed to the article and approved the submitted version.

## Conflict of interest

The authors declare that the research was conducted in the absence of any commercial or financial relationships that could be construed as a potential conflict of interest.

## Publisher’s note

All claims expressed in this article are solely those of the authors and do not necessarily represent those of their affiliated organizations, or those of the publisher, the editors and the reviewers. Any product that may be evaluated in this article, or claim that may be made by its manufacturer, is not guaranteed or endorsed by the publisher.
